# Survivorship of *Anopheles gambiae* sensu lato in irrigated sugarcane plantation scheme in Ethiopia

**DOI:** 10.1186/s13071-021-04630-8

**Published:** 2021-03-06

**Authors:** Dawit Hawaria, Solomon Kibret, Assalif Demissew, Arega Tsegaye, Denekew Bitew, Guiyun Yan, Delenasaw Yewhalaw

**Affiliations:** 1 Yirgalem Hospital Medical College, Yirgalem, Ethiopia; 2 Department of Medical Laboratory Sciences, College of Medicine and Health Sciences, Ambo University, Ambo, Ethiopia; 3 Program in Public Health, University of California at Irvine, Irvine, CA 92697 USA; 4 Department of Medical Laboratory Sciences and Pathology, Institute of Health, Jimma University, Jimma, Ethiopia; 5 Department of Biology, Collage of Natural Science, Jimma University, Jimma, Ethiopia; 6 Tropical and Infectious Diseases Research Center (TIDRC), Jimma University, Jimma, Ethiopia; 7 Department of Statistics, College of Science, Bahir Dar University, Bahir Dar, Ethiopia

**Keywords:** Malaria, Survivorship, *Anopheles gambiae* s.l., Land cover, Sugarcane, Irrigation, Ethiopia

## Abstract

**Background:**

To ensure food security, sub-Saharan Africa has initiated massive water resource development projects, such as irrigated agriculture, in recent years. However, such environmental modifications affect the survivorship and development of mosquitoes, which are vectors of different diseases. This study aimed at determining the effects of irrigation practices on development and survivorship of *Anopheles gambiae* s.l. in Ethiopia.

**Methods:**

A life table experiment was conducted to examine the effect of environmental modification on survivorship of both immature and adult *An*. *gambiae * s.l. in irrigated and non-irrigated areas. The pupation rate and development time of the immatures and adult longevity and fecundity were compared between the two settings.

**Results:**

The estimated mean survival time of female *An. gambiae* s.l. in the irrigated and non-irrigated areas was 37.9 and 31.3 days, respectively. A survival analysis showed that adult females of *An. gambiae* s.l. placed in an irrigated area lived significantly longer than those in a non-irrigated area (*χ*2 = 18.3, *df* = 1, *P* <0.001), and *An. gambiae* s.l. females lived significantly longer than males in both areas (*P *< 0.001).

**Conclusions:**

Adult *An. gambiae* s.l. survivorship was found to be enhanced in the irrigated area compared to non-irrigated area. Longer survival of adult mosquitoes in irrigated areas could have important implications for vectorial capacity and hence malaria transmission.
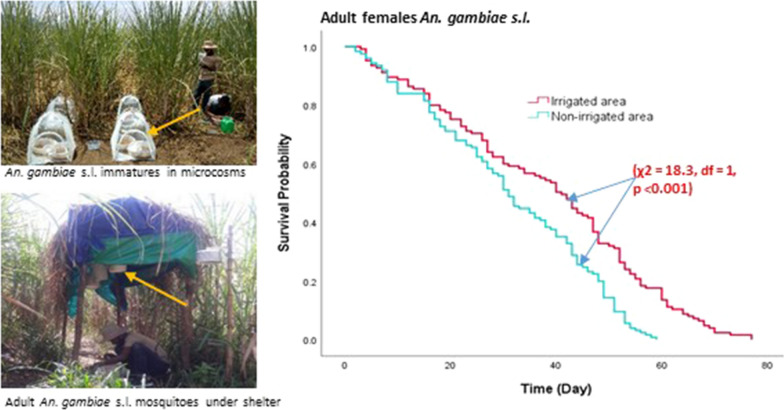

## Background

Mosquito survivorship is an important factor that determines vectorial capacity and malaria transmission potential [1]. For example, the *Anopheles* mosquito needs to survive beyond the extrinsic incubation period of the *Plasmodium* parasites to be able to transmit malaria; the longer a mosquito lives, the higher the number of bites it may inflict [2]. The malaria vector immature survivorship and enhanced larval-to-pupal development rate increase adult population density, which in turn affects the vectorial capacity of mosquito populations in a particular setting [3, 4].

Mosquito survivorship and development may be affected by environmental factors. Temperature (both water and ambient), relative humidity, rainfall, and nutrient availability are key environmental factors governing the dynamics of malaria vectors including development and survival [4–6]. These factors can be strongly influenced by variation in land use and land cover change such as the vegetation cover, landscape, and distance to water bodies [7, 8]. Zhong et al. [9] and Wang et al. [10] reported enhanced survivorship and development of both adults and larvae of *An. sinensis* and *An. minimus*, major malaria vectors in China with higher ambient temperature due to land use and land cover change. Fine-scale variation in the microclimate across different landscapes shapes variation in mosquito population dynamics [11].

In an effort to avert poverty, developing countries have been implementing water resource development projects such as hydropower dams and agricultural development irrigation schemes [7, 12, 13]. Previous studies indicated that such changes in land use and land cover have increased malaria transmission by proliferating vector breeding sites and changing the microclimate that governs the dynamics of the vectors [7, 13–19].

Ethiopia, a country where > 75% of the total area is malarious [20], has been experiencing a massive change in land use and land cover through water resource development projects including irrigation schemes and hydroelectric power dam projects [21]. The Arjo-Dedessa sugar development project site is among the mega-irrigation schemes with an irrigated area covering approximately 4000 ha, with future expansion plans for 80,000 ha, to supply a state-owned sugar factory [22]. The area has historically been a wildlife sanctuary. Long ago, the government settled residents evacuating from other drought-prone areas of the country to establish their lives through subsistence farming. The area is endemic to malaria [22]. A recent entomological study in the same study site demonstrated higher malaria vector breeding habitat diversity, larval occurrence, and abundance in the irrigated area than in the non-irrigated area [23]. However, how this massive environmental modification has been influencing the survivorship and development of major malaria vectors in the area is not yet understood. Understanding malaria vector bionomics in relation to environmental modification helps to model malaria transmission for better evidence-based interventions, which will have a profound effect on realizing the country’s malaria elimination goal by 2030 [24]. We hypothesized that land use and land cover changes, especially massive irrigated agricultural areas, alter the survivorship and development of malaria vectors in the areas.

Therefore, the objective of this study was to determine the effects of an irrigated sugarcane plantation scheme on the development and survivorship of *An. gambiae* s.l. Knowledge of the vector response to environmental modification will give a better understanding of malaria transmission dynamics, which is useful for predicting the impact of environmental modification on malaria transmission intensity and will help establish tailored vector control interventions.

## Methods

### Study setting and period

The study was conducted at the Arjo-Dedessa irrigation development site (8°41'60''N, 36°23'60''E), Southwest Ethiopia, from August to October 2019. Extensive irrigated agriculture represents the most important environmental change in the area. The irrigation development areas were covered with massive irrigated sugarcane plantation (hereafter irrigated area), whereas the surrounding areas were covered with other non-irrigated field crops commonly cultivated in the area (hereafter non-irrigated area). Local communities in the area depend on subsistence farming with practicing smallholder non-irrigated cultivation of mixed crops and cereals. The common crops and fruit trees grown in the area include corn, maize, peanut, sorghum, rice, wheat, coffee, and mango.

### Site selection

For the study, we selected two land use and land cover types: areas covered with irrigated sugarcane plantation and areas covered with other field crops common in the area.

### *Anopheles gambiae* s.l. immature survivorship

#### Adult mosquito collection and larva hatching

Blood-engorged *An. gambiae* s.l. were collected from inside houses and animal shelters in the study area using a mouth aspirator. All collected mosquitoes were kept in paper cages at a field insectary. An oviposition substrate of Petri dishes lined with filter paper disks on moistened cotton wool was kept inside each cage for egg laying. Collected eggs were allowed to hatch, and newly hatched first instar larvae were used for the experiment.

#### Experiment

**Fig. 1:**
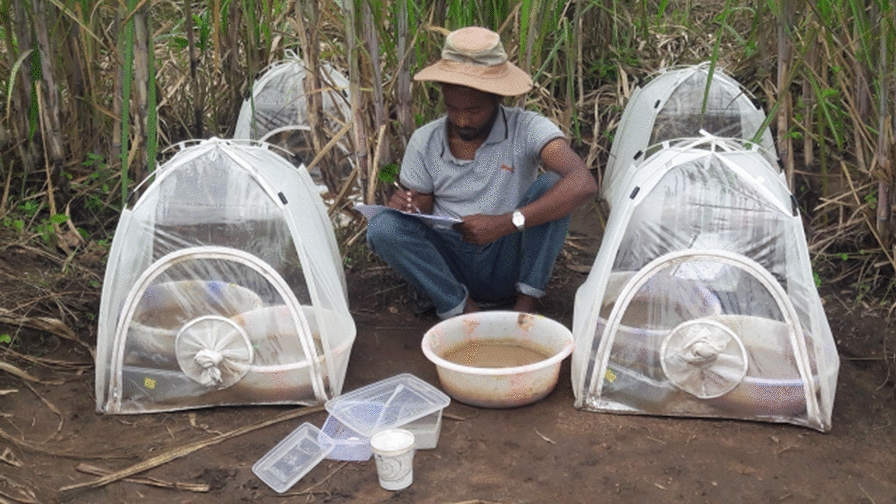
Insect-proof BugDorm tent with washbasins inside

Plastic washbasins (34 cm × 14 cm) were used to imitate natural larval breeding habitats. The washbasins were exposed to an outdoor environment for a week prior to the initiation of the experiment for acclimatization. Then, 2 l of rainwater and 1 kg of soil from the same area were added to each washbasin and left for a day. The washbasins were kept at each selected site in the two different areas (irrigated and non-irrigated areas). Fifty newly emerged first instar *An. gambiae* s.l. larvae were transferred into each washbasin with eight replicates for each site. The water level in the washbasins was checked daily and maintained by adding water if needed. To prevent other insects from invading the washbasins or other mosquitoes from laying eggs, the washbasins were placed inside an insect-proof 61 × 61 × 61 cm^3^ BugDorm tent [BioQuip, Rancho Dominguez, (BD2120), CA, USA] (Fig. 1). All sides of the BugDorm tent were made of clear polyester netting materials so that sunlight was not blocked. The homogeneity of washbasins had an advantage over the natural habitats, which were highly variable in habitat size, larval food conditions (e.g. organic matter), vegetation coverage, light shade, competitors, and predators. Each day the number of surviving larvae, their developmental stage, and mortality were recorded. Pupae were counted and removed daily. Removed pupae were collected in a waterproof paper cup for adult emergence.

### *Anopheles gambiae* s.l. adult survivorship experiment

**Fig. 2:**
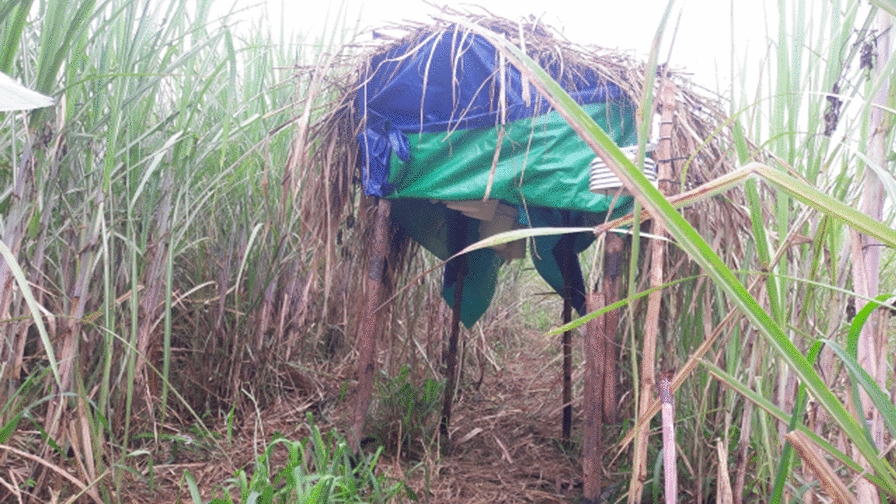
Roof structure from which cages with adult *An. gambiae* s.l. mosquitoes were suspended

In this experiment, *An. gambiae* s.l. adults that emerged from the larval survivorship experiments were used. Within 24 h post-emergence, 25 female and 25 male adult mosquitoes were transferred into a paper cage (21.5 cm × 9 cm). The cages were covered with nylon mesh to prevent mosquito escape. Then, the cages were placed in the irrigated and non-irrigated areas in five replicates for each site. Mosquito cages were hung from the roof structures of small temporary shelters (2 m high) constructed for the purpose of the experiment for rain protection (Fig. 2). To prevent ants from reaching and scavenging the mosquitoes, grease was applied to the suspension twines. Mosquitoes were provided with 10% sucrose solution and a bloodmeal from a human arm (DH) every other day for 20 min. An oviposition substrate consisting of a Petri dish lined with a filter paper disk on moistened cotton wool was placed for egg-laying. The oviposition substrate in each cage was examined daily for the presence of eggs, and the number of eggs laid was examined under a dissecting microscope, counted, and recorded. The cages were examined daily for the numbers of surviving and dead mosquitoes. The dead mosquitoes were recorded and removed from the cage daily.

### Microclimate data collection

For the larval survivorship experiment, a HOBO data logger (Onset Computer Corp., MX2202, Bourne, MA) was placed in each washbasin, 1 cm below the water surface, and then hourly water temperature and light intensity were recorded for the entire duration of the experiment. For the adult survivorship experiment, HOBO data loggers (Onset Computer Corp., MX2301) were kept close to the experiment set-up 2 m above ground, and then the hourly ambient temperature and relative humidity were recorded for the entire duration of the experiment.

### Data analysis

The pupation rate of *An. gambiae* s.l. larvae was calculated as the proportion of first instar larvae that developed into pupae. Mean larval-to-pupal development time was calculated. Stage-specific larval development time and mortality rate were calculated. Kaplan-Meier survival analysis was performed to determine the variation in mean daily survivorship of mosquitoes placed in two different land use and land cover areas. A log-rank test was used to determine the difference between two survival curves. Daily average, minimum, and maximum temperatures, relative humidity, and light intensity were calculated from the hourly record data to determine the effect of different land uses and land covers on the microclimate of local niches where mosquitoes were tested for survivorship. Independent sample t-test was performed to compare mean pupation rate, development time, and microclimate differences across irrigated and other non-irrigated crop areas. The analysis was performed using IMB SPSS Statistics 25, R 3.5.2, and Microsoft Excel 2016.

## Results

Around 300 blood-engorged *An. gambiae* s.l. were collected from indoors and outdoors (cow shelter) using mechanical mouth aspirators. Eight hundred first instar larvae hatched from the field-collected mosquitoes were used for the experiments in irrigated and non-irrigated areas, 400 each.

### Developmental time and survivorship of *An. gambiae* s.l. larvae

**Fig. 3:**
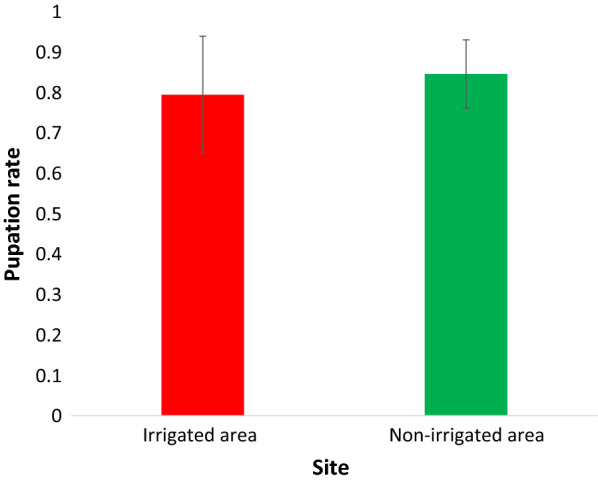
Pupation rate of *An. gambiae* s.l. larvae in irrigated and non-irrigated areas, Southwest Ethiopia, 2019

**Fig. 4:**
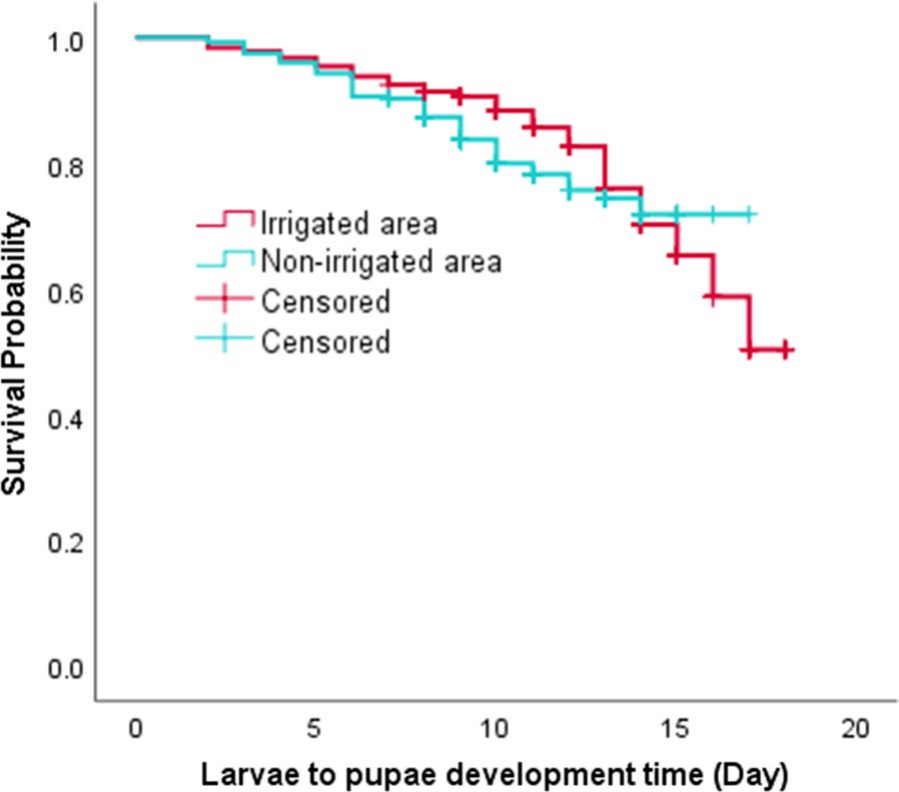
Survivorship of *An. gambiae* s.l. immatures in irrigated and non-irrigated areas, Southwest Ethiopia, 2019

The proportion of larvae that completed development from first instar larvae to pupae in the irrigated area and non-irrigated area was 79.4% (95% CI 0.66–0.93) and 84.5% (95% CI 0.77–0.92), respectively. Statistical analysis showed that the difference in pupation rate was not significant between the irrigated and non-irrigated area (*t* = 2.22, *P* = 0.208) (Fig. 3). The mean larval-to-adult development time of *An. gambiae* s.l. larvae in the irrigated and non-irrigated areas was 12.5 and 12, respectively. Similarly, the median larvae-to-pupae development time in the irrigated area was 12.5 (95% CI 10.2–14.8) days and in the non-irrigated area 12 (95% CI 9.7–14.2) days (Table 1). Kaplan-Meier survival analysis showed no significant difference in larval survivorship between the two areas (*χ*^2^ = 2.62, *P* = 0.106) (Fig. 4).

**Table 1:** Means and medians of survival time for immature *An. gambiae* s.l. in irrigated and non-irrigated areas, Southwest Ethiopia, 2019

Site	Mean with 95% CI	Median with 95% CI	Overall comparisons		
Irrigated area	12.5 (10.3–14.4)	12.5 (10.2–14.8)	*χ*^2^	*df*	*P*-value
Non-irrigated area	12.1 (11.6–13.9)	12.0 (9.7–14.2)	2.62	1	0.106

Stage-specific survival and mortality analysis showed a slight increment in the mortality rate as the larvae developed to proceeding larval instars in both settings (Table 2).

**Table 2:** Stage-specific survivorship and mortality rate of immature *An. gambiae* s.l. in the irrigated area, Southwest Ethiopia, 2019

Stage	Irrigated area			Non-irrigated area		
	Development time (day)	Cumulative survival rate	Stage mortality rate	Development time (day)	Cumulative survival rate	Stage mortality rate
1st Instar	2.3	0.98	0.02	2.1	0.98	0.03
2nd Instar	2.5	0.96	0.03	2.4	0.95	0.03
3rd Instar	4.6	0.92	0.04	3.5	0.89	0.06
4th Instar	5.1	0.79	0.19	4.5	0.84	0.09

### Adult *An. gambiae* s.l. survivorship and fecundity

**Fig. 5:**
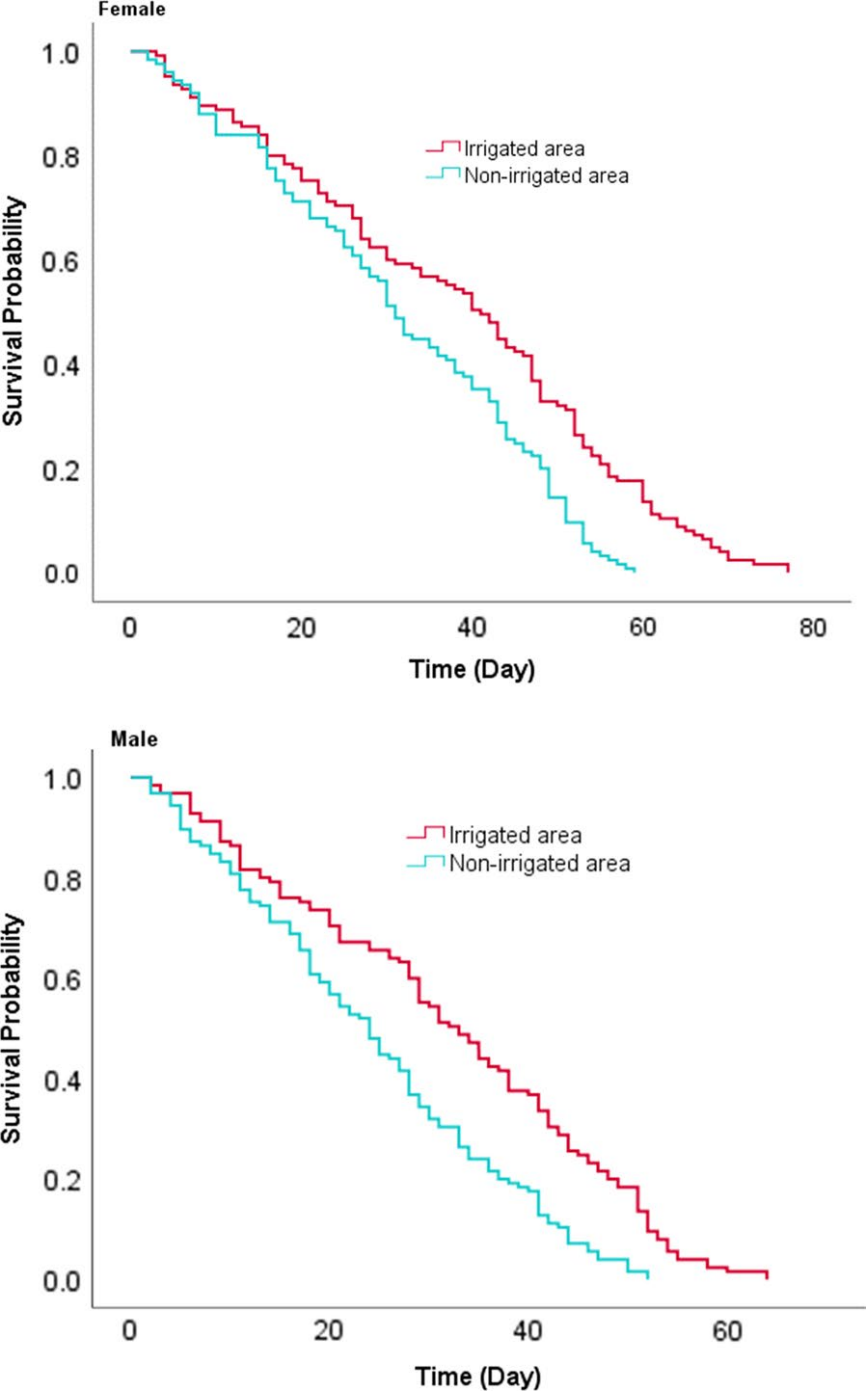
Survivorship of adult *An. gambiae* s.l. in irrigated and non-irrigated areas, Southwest Ethiopia, 2019

Survival analysis showed that female *An. gambiae* s.l. placed in the irrigated area survived significantly longer than those in the non-irrigated area (*χ*^2^ = 18.3, *df* = 1, *P* < 0.001) (Fig. 5).

The estimated mean survival time of female *An. gambiae* s.l. in irrigated and non-irrigated areas was 37.9 and 31.3 days, respectively (Table 3). Again, female mosquitoes showed the higher median survival period (41.0 days) in irrigated than non-irrigated area (31.0 days). A similar result was found in that male *An. gambiae* s.l. survived a longer period in the irrigated than non-irrigated area (*χ*^2^ = 23.1, *df *= 1, *P* <0.001) with mean survival time of 31.8 and 24.2 days, respectively (Table 3). The median survival period for male mosquitoes was 33.0 days in the irrigated area and 24.0 days in the non-irrigated area (Table 1). Male *An. gambiae* s.l. survival was decreased compared to that of females in both the irrigated (*χ*^2^ = 14.9, *P* < 0.001) and non-irrigated areas (*χ*^2^ = 20.9, *P* < 0.001) (Additional file 1).

**Table 3:** Means and medians of survival time for adult *An. gambiae* s.l. in irrigated and non-irrigated areas, Southwest Ethiopia, 2019

Site	Female *Anopheles* gambiae		Male *Anopheles* gambiae	
	Mean with 95% CI	Median with 95% CI	Mean with 95% CI	Median with 95% CI
Irrigated area	37.9 (34.8–41.5)	41.0 (35.9–46.1)	31.8 (28.9–34.7)	33.0 (28.3–37.7)
Non-irrigated area	31.3 (28.5–34.1)	31.0 (27.9–34.1)	24.2 (21.8–26.6)	24.0 (20.3–27.6)

Of 7737 eggs laid by the female mosquitoes throughout the experiment period, 5125 (66.2%) were from the mosquitoes placed in the irrigated area and 2612 (33.8%) were from mosquitoes in the non-irrigated area. The study showed that fecundity of mosquitoes was 96.2% higher in the irrigated area (80 eggs/day) than in the non-irrigated area (average 33 eggs/day). The mean number of eggs laid was (41 ± SE 11.63 eggs/mosquito) and (21 ± 5.61 eggs/mosquito) in the irrigated and non-irrigated area, respectively. Statistical analysis showed that the difference in fecundity was significant between the irrigated and non-irrigated area (*t* = 2.83, *P* = 0.002) (Additional file 2).

### Aquatic habitat microclimate during larval survivorship experiment

**Fig. 6:**
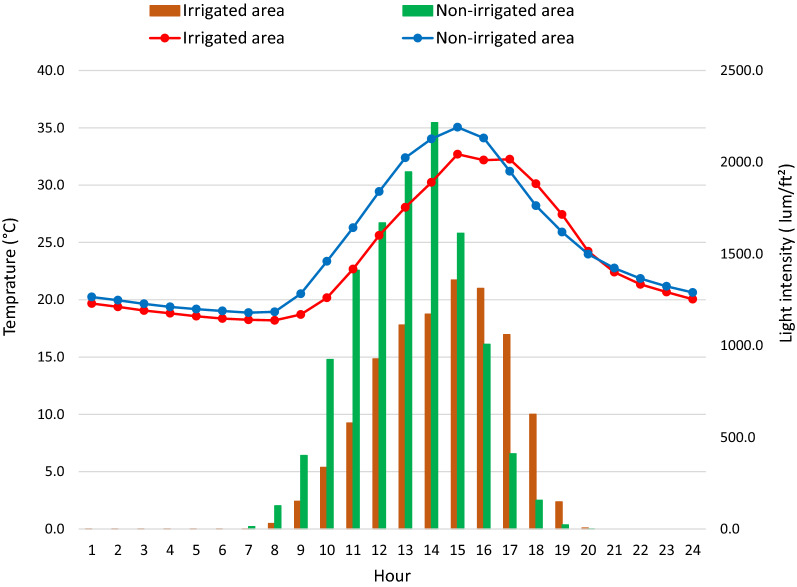
Mean hourly temperature and light intensity 24-h daily cycle in washbasins in irrigated and non-irrigated areas, Southwest Ethiopia, 2019

An independent sample t-test analysis on microclimate differences between the two study settings indicated that mean hourly water temperature (°C) in washbasins placed at the non-irrigated area was 1.1 °C higher than in washbasins in the irrigated area (*t* = − 2.85, *P* = 0.004). Similarly, mean light intensity (lum/ft2) in the non-irrigated area (mean = 497.4 ± 982.2) was significantly higher than in the irrigated area (mean = 372.7 ± 664.8), (*t* = − 2.47, *P* = 0.014) (Table 4 and Fig. 6). Mean maximum and minimum temperature and light intensity were also significantly higher in washbasins in the non-irrigated area compared to the irrigated area (Table 4).

**Table 4:** Mean hourly temperature and light intensity in washbasins in the irrigated and non-irrigated areas, Southwest Ethiopia, 2019

Microclimate	Irrigated area (M ± SE)	Non-irrigated area (M ± SE)	*t*	*df*	*P*
Mean temperature (°C)	23.3 ± 5.7	24.4 ± 6.3	− 2.85	1068	0.004
Mean maximum temperature (°C)	24.4 ± 6.5	25.4 ± 7.2	− 2.53	1068	0.012
Mean minimum temperature (°C)	22.5 ± 5.1	23.4 ± 5.4	− 2.83	1068	0.005
Mean light intensity (lum/ft^2^)	372.7 ± 664.8	497.4 ± 982.2	− 2.47	1068	0.014
Mean maximum light intensity (lum/ft^2^)	713.0 ± 1311.7	931.0 ± 1698.0	− 2.28	1068	0.018
Mean minimum light intensity (lum/ft^2^)	174.4 ± 311.9	229.1 ± 495.5	− 2.21	1068	0.027

*M ± SE: mean ± standard error*

### Ambient microclimate during adult survivorship experiment

**Fig. 7:**
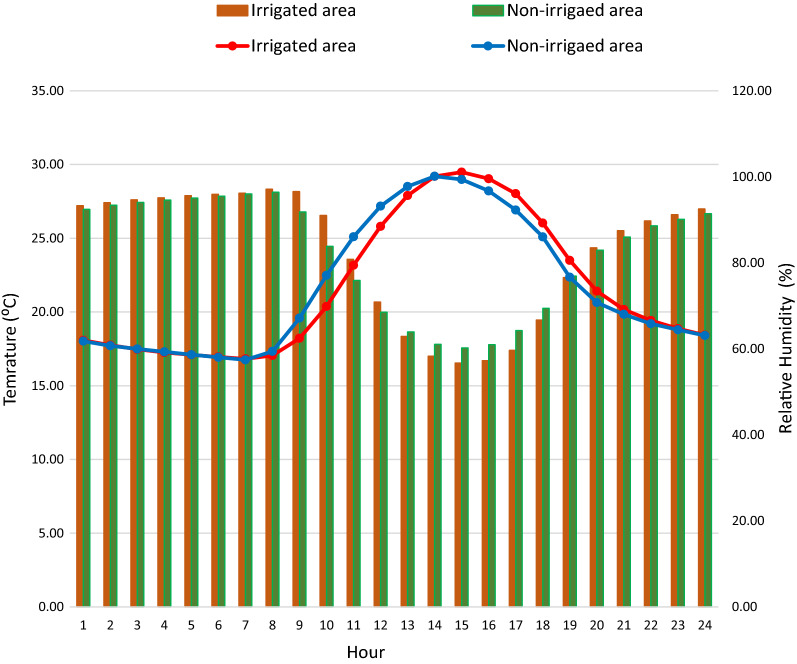
Mean hourly ambient temperature and relative humidity 24-h daily cycle in irrigated and non-irrigated areas, Southwest Ethiopia, 2019

There was no significant difference in ambient hourly average, maximum and minimum temperature and relative humidity between the irrigated area and non-irrigated area. However, mean light intensity between the two sites was different (*P* = 0.001) (Table 5 and Fig. 7).

**Table 5:** Hourly microclimate condition of mosquito niches in irrigated and non-irrigated areas, Southwest Ethiopia, 2019

Microclimate	Irrigated area (M ± SE)	Non-irrigated area (M ± SE)	*t*	*df*	*P*
Mean temperature (°C)	21.56 ± 4.80	21.60 ± 4.81	− 0.26	3176	0.790
Mean maximum temperature (°C)	22.22 ± 5.09	22.24 ± 5.10	− 0.09	3176	0.927
Mean minimum temperature (°C)	20.90 ± 4.56	20.92 ± 4.56	− 0.12	3176	0.904
Mean relative humidity (%)	82.65 ± 15.73	82.30 ± 14.58	− 0.63	3176	0.522
Mean maximum relative humidity (%)	86.11 ± 13.77	86.55 ± 11.80	− 0.92	3176	0.339
Mean minimum relative humidity (%)	78.95 ± 17.78	78.12 ± 17.23	1.31	3176	0.187
Mean light intensity (lum/ft^2^)	324.3 ± 517.5	709.0 ± 1242.3	− 11.7	2952	0.001
Mean maximum light intensity (lum/ft^2^)	571.7 ± 982.5	1106.8 ± 1834	− 10.3	2952	0.001
Mean minimum light intensity (lum/ft^2^)	180.1 ± 267.5	366.6 ± 663.4	− 10.7	2952	0.001

*M ± SE: mean ± standard error*

## Discussion

In this study, we investigated the effects of environmental modification on the development, survivorship, and fecundity of malaria vector mosquitoes. We hypothesized that irrigated sugarcane plantation areas enhance development, survivorship, and fecundity compared to non-irrigated field crop areas because of better microclimates and nutrients following environmental modification. However, the study showed no significant difference in development and survivorship of *An. gambiae* s.l. immatures between the two areas.

Variation in vegetation cover may affect the radiation flux and energy balance off the land surface and thus may modify the microclimate [25]. By the time the experiment was conducted, sugarcane plantation was at its maturity stage, which is dense and leafy, which could partly limit direct sunlight from reaching the washbasins, whereas in the surrounding crops the field areas were relatively less dense. The mean hourly water temperature in the non-irrigated area increased by 1.1 °C compared to the irrigated area. This could partly explain the observed 5.1% greater pupation rate in the non-irrigated area compared to the irrigated area. Studies reported elsewhere indicated that increased temperature due to land use and land cover increased the larval survival rate [10, 26–30]. Tuno et al. [29] reported that the survivorship of *An. gambiae* larvae was reduced from 56% in habitats fully exposed to sunlight to 1.5% in habitats with forest canopy in western Kenya. Wang et al. [10] also reported the pupation rate of *An. minimus*, a malaria vector in China, to be 52.5%, 12.5%, and 3.8% in the deforested, banana plantation, and forested areas, respectively, which is far lower than our findings of 79.4% and 84.5% at the irrigated and non-irrigated areas, respectively.

Nutrient availability may affect the survival, pupation rate, and development time. The potential food source of anopheline larvae may include but not be limited to bacteria, fungi, debris, and organic matter. The abundance and structure of microbes such as algae and photosynthetic cyanobacteria in aquatic habitats may have changed in response to land use and land cover [31, 32]. Organic matter and debris in the soil at different settings may not be the same, which could possibly vary with changes in the surrounding land use and land cover. Kebede et al. [33] reported that maize pollen provides nutrition for larval anopheline mosquitoes showing that the incidence of malaria was about ten times higher in high maize cultivation areas. In our case, the debris of sugarcane plantation and other field crops might not be the same but the result showed both areas support mosquito development, which needs further investigation of soils' biological and chemical composition in relation to immature mosquito development.

The higher pupation rate and longer survivorship of *An. gambiae* s.l. immatures generally could increase vectorial capacity to enhance malaria transmission. Based on these findings alone, we cannot conclude that the irrigated area encounters less or equal malaria risk compared to the surrounding environs. Recently, in a study conducted from the same area, significantly more diverse breeding sites and larva abundance have been reported in irrigated sugarcane plantation areas than in their surroundings [23]. Thus, more diversified breeding sites with a 79.4% pupation rate could certainly outweigh the malaria burden over surrounding environs with less habitat diversity and relatively the same pupation rate.

Adult *An. gambiae* s.l. placed in the irrigated area survived longer than those in a non-irrigated area. Adult female mosquitoes survived longer than males in both settings. Our findings of mosquito longevity were in line with previous studies elsewhere. For instance, Okech et al. [34], reported mean survival of 33 days for *An. gambiae* s.l. in western Kenya, which is 6 days shorter than our finding. Gary and Forster [35] found that *An. gambiae* s.l. mosquitoes had a median survival time of 29 days under insectary conditions, but in our study, the median survival time for female *An. gambiae* s.l. was 41 and 31 days in the irrigated and non-irrigated area, respectively. The longer survival of mosquitoes in the irrigated area indicates that *An. gambiae* s.l. is well adapted to the environmental conditions. Enhanced survival of malaria vectors is among the determinants of increased mosquito vectorial capacity [36]. A long life of an adult female mosquito increases her opportunities to encounter an infected human host and the extrinsic incubation period of malaria parasites so that they can reach the salivary glands after an infective bloodmeal and be transmitted in later bloodmeals to uninfected hosts [1, 3, 37]. This has implications for malaria transmission at the locality.

The experimental set-up at both study settings were the same, and human blood and sugar were provided in a similar way. Thus, the only difference was the environment where the experiments were situated, being an irrigated and non-irrigated area. There was no significant difference in mean, maximum, and minimum hourly ambient temperature and relative humidity between the two environments. Previous studies indicated that *An. arabiensis*, a primary vector in Ethiopia, generally prefers areas with low humidity and high temperature [38]. A similar study also demonstrated that reduced humidity and increased temperatures following deforestation create a more suitable environment for adult *An. arabiensis* to survive longer [26]. Therefore, in our study setting the determinants involved in supporting better survival of adult *An. gambiae* s.l. at the irrigated area warrants further investigation.

The average daily fecundity of *An. gambiae* s.l. mosquitoes in the irrigated area was 96.2% higher compared to the non-irrigated area. Increased survival together with enhanced fecundity of malaria vectors in the irrigated area suggests that the longevity and biotic potential of *An. gambiae* s.l. in the area are very high, favoring increased population density, and thus the species could contribute greatly to malaria transmission. Better survival and fecundity in the irrigated area in our study are in agreement with the study conducted in Ethiopia at the laboratory level demonstrating that gravid *An. arabiensis* females were attracted to sugarcane pollen volatiles [39].

This study had several limitations. The experiment was done at one time point of the maturity stage of the irrigated sugarcane area. The microclimate conditions in the irrigated area during the seedling/germinating stage, tillering stage, grand growth stage, and maturity stage [40] could not be the same, which in turn influenced the mosquito survivorship. Information on the chemical and nutrient composition of the soil used as a substrate was not included in the study. Moreover, the experiments were conducted under controlled conditions for all potential biological factors that may influence mosquito survival, such as predators and competitors, which might lead to an overestimation of the survival time compared to the actual time.

## Conclusion

Irrigated sugarcane plantation significantly enhances the survivorship and fecundity of adult *An. gambiae* s.l., the major malaria vector in Ethiopia. The study results on survivorship parameters of malaria vector mosquitoes under a variety of environmental conditions are helpful to model the impact of environmental modification on vector population dynamics and help devise tailor-made vector control strategies. Moreover, longer survivorship of adult mosquitoes in irrigated areas calls for larval management to reduce the vector population and subsequent malaria transmission.

## Supplementary Information

**Additional file 1:** Survivorship comparison of adult male and female *An. gambiae* s.l. in irrigated and non-irrigated areas, Southwest Ethiopia, 2019.

**Additional file 2:** Mean eggs laid per mosquito in irrigated and non-irrigated areas, Southwest Ethiopia, 2019.

## Data Availability

The datasets used and/or analysed during the current study are available from the corresponding author on reasonable request.
