# Supplementary figures and images for: Survivorship of Anopheles gambiae sensu lato in irrigated sugarcane plantation scheme in Ethiopia
Source: Parasit Vectors. 2021 Mar 6;14:142. doi: 10.1186/s13071-021-04630-8 (PMC7936430; doi:10.1186/s13071-021-04630-8)

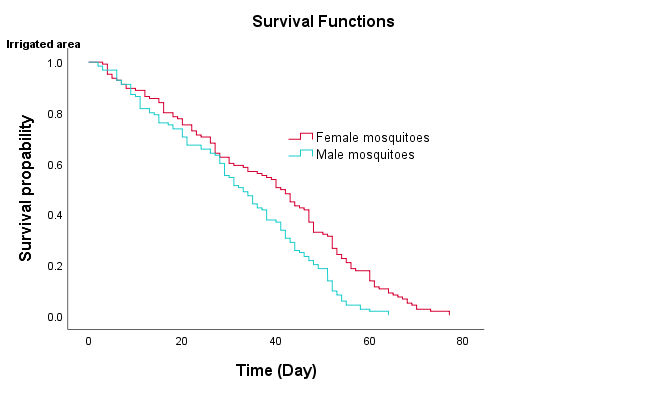


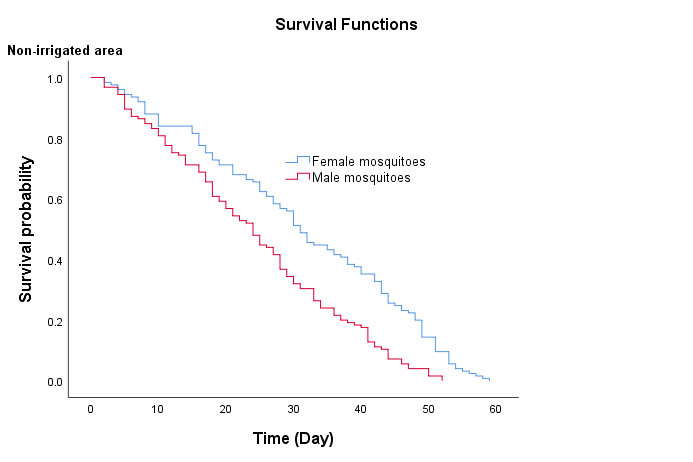


**Supplementary file 1**

Supplement: Supplementary file 1 — Additional file 1: Survivorship comparison of adult male and female An. gambiae s.l. in irrigated and non-irrigated areas, Southwest Ethiopia, 2019. [file 13071_2021_4630_MOESM1_ESM.docx]
