# Supplementary material for: Survivorship of Anopheles gambiae sensu lato in irrigated sugarcane plantation scheme in Ethiopia
Source: Parasit Vectors. 2021 Mar 6;14:142. doi: 10.1186/s13071-021-04630-8 (PMC7936430; doi:10.1186/s13071-021-04630-8)
Supplement: Supplementary file 2 — Additional file 2: Mean eggs laid per mosquito in irrigated and non-irrigated areas, Southwest Ethiopia, 2019. [file 13071_2021_4630_MOESM2_ESM.docx]

**Supplementary file 2** Mean eggs laid per a mosquito in irrigated and non-irrigated areas, Southwest Ethiopia, 2019.

| Statistics | Irrigated area | Non-irrigated area | T Stat | P (T<=t) |
| --- | --- | --- | --- | --- |
| Mean | 41.0 | 21.0 | 2.83 | 0.002 |
| Standard Error | 11.6 | 5.6 |  |  |
| Median | 44.7 | 14.8 |  |  |
| Standard Deviation | 26.0 | 12.5 |  |  |
| Minimum | 28.5 | 0 |  |  |
| Maximum | 96 | 34 |  |  |
| Confidence Level (95%) | (20.0 – 84.5) | (0 – 32.2) |  |  |
